# Natural Products for Drug Discovery in the 21st Century: Innovations for Novel Therapeutics

**DOI:** 10.3390/molecules28093690

**Published:** 2023-04-25

**Authors:** Ericsson Coy-Barrera, Ifedayo Victor Ogungbe, Thomas J. Schmidt

**Affiliations:** 1 Bioorganic Chemistry Laboratory, Military University Nueva Granada, Nueva Granada Campus, Cajicá 250247, Colombia; 2 Department of Chemistry, Jackson State University, Jackson, MS 39217-0095, USA; 3 Institute of Pharmaceutical Biology and Phytochemistry (IPBP), University of Münster, PharmaCampus-Corrensstrasse 48, D-48149 Münster, Germany

## 1. Introduction

Natural products (NPs) from plants, fungi, animals, and microorganisms have historically played important roles in drug discovery. Due to their vast naturally occurring chemodiversity and structural complexity, research into NPs has uncovered bioactive agents, hits, and lead compounds which promote pharmacological advances to treat different health problems, mainly related to cancer, infectious diseases, inflammation, pain, and metabolic and cardiovascular disorders [1]. In recent decades, NP-inspired drug discovery has experienced a leap in progress due to several innovations overcoming crucial barriers and limitations to developing NP-derived drugs [2]. In addition, the innovations are also oriented toward expanding NP chemical space by identifying more NPs and derivatives, and designing and optimizing bioactive NP-based libraries to find novel therapeutic agents with low toxicity and good pharmacological performance [3]. Such a rise has been possible due to technological advances based on analytical techniques, (micro)fractionation approaches, precision bioassays, high-throughput screening, omics-mediated strategies, and computational techniques and data processing [4], which have assisted NP research for drug discovery in the 21st century to multidimensional innovations for novel therapeutics. In this regard, an increasing number of highly promising and innovative discoveries in this field are published in NP periodicals such as Molecules every year.

Aware of this attractive panorama in modern NP research, we launched this Special Issue within the Natural Products Chemistry Section of *Molecules*, entitled “Natural Products for Drug Discovery in the 21st Century: Innovations for Novel Therapeutics”. The scope of this Special Issue was oriented to feature some of the recent and most promising findings in this area, involving a clear connection between NP chemical structure and biological/therapeutic effects through the development and application of new strategies and approaches for NP discovery, as well as timely, critical, and comprehensive reviews on particularly interesting topics within the broad field of the chemistry and biological activity of NPs. Hence, this Special Issue covers twelve contributions comprising eight original research manuscripts and four reviews. The guest editors compiled an overview of these contributions, and the main findings are briefly described below. 

## 2. Main Findings

Cancer is still a current concern that has been widely addressed through the investigation of bioactive NPs [5], and, therefore, several studies compiled in this Special Issue are related to antiproliferative NPs against several cancer cell lines. In this context, Reda et al. studied the Egyptian cytotoxic plant *Centaurea lipii*. They compared the LC-MS/MS-derived chemical profiles of extracts obtained from the aerial parts of eight *Centaurea* species using feature-based molecular networking (FBMN) [6]. The global overview of the test *Centaurea* metabolite profiles involved 81 annotated metabolites, recognizing various compounds that uniquely occurred in the cytotoxic plant (i.e., *C. lipii*). The bio-guided fractionation of *C. lipii* led to the isolation of cynaropicrin, a sesquiterpene lactone with a promising cytotoxic activity against the CCRF-CEM leukemia cell line (IC_50_ = 1.82 µM). Using a similar approach, Diaz et al. employed an integrative study on antiproliferative activity against three human cancer cell lines (i.e., PC-3, SiHa, and A549) and the LC-MS chemical profiles of seed extracts from ten accessions of *Genista monspessulana* [7]. Seven compounds were recognized as responsible for the perceived activity through multiple covariate statistics (15.8 µM < IC_50_ < 34.3 µM). The most active compounds (i.e., (–)-cytisine and alpinumisoflavone, IC_50_ < 18.6 µM) were combined to create a cytisine-linked isoflavonoid (CLIF). This CLIF exhibited better activity against PC-3 (IC_50_ = 10.1 µM) and even the other two cancer cell lines (i.e., SiHA and A549, IC_50_ = 17.5 and 46.8 µM, respectively). The findings described in these two studies indicate that the implemented composition–activity associative strategies to select relevant, unique features from active plant extracts is part of the innovative approaches used within the last decade to find and guide the targeted isolation of active compounds for drug discovery.

Other NP research-related innovations are oriented to search for novel therapeutic alternatives against emergent cancer diseases or produce semisynthetic derivatives with better activity from abundant NPs. In the first case, Peraza-Labrador et al. investigated the antiproliferative activity of *Lycium barbarum* fruits against oral and oropharyngeal HPV16 squamous cell carcinoma (OSCC), a devastating disease with high and increasing incidence in recent years [8]. The *L. barbarum*-derived phenolic-rich extract inhibited the SCC090 and CAL27 cell lines, involving cell cycle arrest and apoptosis induction. The presence of flavonols/flavan-3-ols and tyramine-conjugated hydroxycinnamic acid amides was linked with the observed effects. This association rationalized a plausible immunomodulatory effect, which requires further studies to define the roles of the active principle (alone or combined). In the second case, Beer et al. studied the antiproliferative activity of three sesquiterpenes and their derivatives (n = 16) against six human solid tumor cell lines [9]. Two oxo-nitrogenated ilicic acid derivatives were the most antiproliferative compounds against test cell lines (i.e., 5.3 μM < GI_50_ < 14 μM). In addition, the 1,2,3-triazole ilicic alcohol derivative also improved the activity (12 μM < GI_50_ < 17 μM). This study also evaluated anti-*T. cruzi* activity, in which an oxo-nitrogenated tessaric acid derivative and a 1,2,3-triazole ilicic alcohol derivative exhibited the best activity and selectivity (IC_50_ < 9.3 µM, and SI > 8.0). The outcome demonstrated that transforming abundant and easily isolable NPs is a useful strategy to expand NP chemical space to search for NP-derived agents with improved activity.

In their early stage anticancer drug discovery investigations, Abd-Alhaseeb et al. [10] studied the effects of evening primrose oil, the selective estrogen receptor modulator tamoxifen, and the combination of both on the growth of breast cancer cells in vitro. Methyl linoleate and methyl palmitate are the chief components of evening primrose oil (EPO). Since combination therapy provides an opportunity to simultaneously deploy multiple mechanisms of action against pathogens and malignant tumors, the authors surmised that EPO potentiated the effect of tamoxifen on MCF-7 and MDA-MB-231 BC breast cancer cell lines. The molecular features of the combination include the induction of apoptosis, inhibition of angiogenesis, and cell cycle arrest.

To identify molecules that can protect the specialized kidney epithelial cells, podocytes, from injury by excessive palmitic acid (PA), such as that observed in diabetic nephropathy, Kaewin et al. [11] screened a library of 355 NPs in a cell viability assay. The group discovered that the fungal metabolites 3-hydroxyterphenyllin (3-HT) and candidusin A (CDA) protected PA-induced podocyte injury at IC_50_ values of 16 and 18 µM, respectively. The authors concluded that oxidative stress is a significant contributor to PA-induced injury to podocytes and that the fungal metabolites could exert antioxidant effects via scavenging free radicals and upregulation of Bcl-2. 

Pilon et al. [12] analyzed essential oils for the similarity of their metabolite spectra using GC-MS-based molecular networking as a cutting-edge data visualization approach with the aim of discovering constituents with larvicidal activity against the disease-spreading mosquito *Aedes aegypti*. They obtained clusters of molecular species called molecular nodes that were theorized to have similar compounds and potentially similar larvicidal activities. The approach allowed the investigators to infer chemotaxonomic relationships between the oils and to prioritize oils containing relatively better activities against larvae of *Aedes aegypti*. The approach suggested that acyclic, monocyclic, and methane monoterpenes have better larvicidal activities in oils.

Ailanthone and bruceine A, two degraded triterpenoids of the quassinoid-type known for their anthelminthic activity, were investigated by Knetzger et al. [13] for their effects on the reproductive tissues of *Caenorhabditis elegans*, a model nematode, using a variety of microscopic techniques, including atomic force microscopy. The investigators showed that the quassinoids affected the development of proximal gonads and meiotic germ cells and caused defects in the nematode’s spermathecae. Ailanthone and bruceine A also appear to interact with nucleic acids in the tissues, and therefore they may be potentially genotoxic. The authors surmised that the impacted tissues could be targeted for discovering and developing quassinoid-based anthelmintics.

It can already be seen from the exciting original articles summarized so far that NPs of various biosynthetic classes and origins continue to show interesting bioactivities with a large potential for exploitation as leads to new drugs. Four review articles round off the Special Issue by providing overviews on particularly promising types of NPs. 

The review by Metibemu and Ogungbe [14] focuses on carotenoids, a class of tetraterpenoids most prominently known from plants as accessory photosynthetic pigments and renowned for their nature as vitamins in the human diet and their antioxidant potential. The article summarizes current knowledge on the major aspects of carotenoid biosynthesis and phytochemistry, as well as their pharmacology. It is exciting to read the current knowledge on the mechanisms underlying all the different bioactivities of carotenoids caused by effects on various important signaling pathways, such as Akt/mTOR, Bcl-2, SAPK/JNK, JAK/STAT, MAPK, Nrf2/Keap1, and NF-κB, which lead to the potential utility of this interesting class of NPs against diseases and conditions ranging from cancer via diabetes, aging and inflammation, ocular, skin, neurodegenerative and cardiovascular disorders.

Cancer certainly remains one of the most challenging health risks to humankind, and it is therefore no wonder that three out of the four reviews [15,16,17] focus entirely on NPs with anticancer activity and potential utility as leads against various neoplastic diseases. It is highly interesting and worth mentioning in this editorial that one particular class of NPs, sesquiterpene lactones (STLs), plays a major role in all three of these reviews. 

Laurella et al. [15] summarize the knowledge on STLs as inhibitors of pancreatic cancer, often associated with mutation of the K-ras oncogene, which these compounds appear to influence by interaction with the NF-κB, MAPK, and PI3K signaling pathways, all involved in tumor survival. Interestingly, most of the investigated STLs, some previously known to have anticancer potential, preferentially affected one or two of the mentioned signaling pathways, while the guaianolide aguerin B from a *Centaurea* (Asteraceae) species interfered with all three of them and thus appears to be a very promising candidate for deeper studies with the aim of developing new therapies for such prostate cancers. Among the compounds mentioned in the review [15] is the STL deoxyelephantopin from the *Elephatopus* species (Asteraceae), which had previously been demonstrated to act against cancer cells. The promising antitumor potential of this compound and its congener isodeoxyelephantopin is the subject of the review by Mehmood and Muanprasad [16]. The authors provide a detailed overview on these compounds’ multi-faceted interference with signaling pathways involved in cancer progression, ultimately leading to apoptosis. Thus, the MAPK and Wnt/β-Catenin, STAT3, NF-κB, and PI3K/AKT/mTOR pathways, as well as the mechanisms of autophagy and tumor invasion/metastasis, are targets of these compounds, and various semisynthetic derivatives are also described. One derivative, DETD-35, with a naphthyl acetic acid ester moiety, is highlighted as particularly promising.

The review by Schmidt and Klempnauer [17] highlights a completely novel mechanism of anticancer action of various NPs, which these authors recently discovered. The proteins MYB, C/EBPβ, and p300 cooperate to form a transcriptional module involved in the expression of genes related to cell differentiation and proliferation, and it has been demonstrated that this activity is relevant for tumorigenesis, e.g., in certain types of leukemia, but also in other tumors. Various NPs inhibiting the activity of this machinery were reported to inhibit the growth of tumor cells and cancer. The review article introduces the various classes of NPs found by the authors to inhibit the interplay between the three proteins by interference with several different target sites. Very interestingly, besides some naphthoquinones, a withanolide, quinonemethide triterpenes, and a few sesquiterpene dialdehydes, a variety of sesquiterpene lactones (STLs) were among these compounds. Thus, e.g., helenalin acetate from *Arnica* species was found to be a very potent inhibitor of the interaction between C/EBPβ and the TAZ2 domain of p300, thereby impairing the function of the transcription module. Investigations on the potential of these compounds have shown their potency as antitumor leads and their anti-inflammatory properties, thus receiving further attention from these discoveries.

## 3. Conclusions

The contributions to this Special Issue demonstrate the huge potential that NPs continue to possess as hits and leads for developing prospective new drugs. Besides a strong focus on the identification and characterization of many new and very promising biologically active NPs against cancer [6,7,8,9,10], infective/parasitic diseases, including their vectors [9,12,13], and diabetic kidney disease [11], it is worth emphasizing that new approaches and methodologies for NP-related drug research are an important focus of this SI. Thus, new methods to identify new bioactive entities in the complex matrices of extracts using hyphenated analytical techniques in combination with computer-aided data analysis tools (e.g., multivariate statistics, molecular networking) are the focus of several articles [6,7,12] utilizing them successfully to identify new bioactive hits. Such techniques provide a holistic evaluation of the active principle(s) in a candidate organism/extract under study in a short time and with little financial effort, so that they are now becoming increasingly important. A new approach to investigating the mechanism of action of bioactive NPs using atomic force microscopy described in the article by Knetzger et al. also deserves special mention due to its high development potential [13]. The reviews within this SI [14,15,16,17], finally, provide a comprehensive source of current knowledge on particularly interesting types of NPs and their bioactivity. Indeed, the discovery of an entirely new mechanism of anticancer action with various NPs targeting it [17] demonstrates that NP research still yields fundamentally new findings in drug research based on the search for promising leads to develop innovative antitumor therapies. 

Overall, the many exciting findings newly described [6,7,8,9,10,11,12,13] and reviewed [14,15,16,17] in this Special Issue can confidently lead us to a simple conclusion: natural products have a very bright future in the drug research of the 21st century!
